# Supplementary material for: Molecular profiling of driver events in metastatic uveal melanoma
Source: Nat Commun. 2020 Apr 20;11:1894. doi: 10.1038/s41467-020-15606-0 (PMC7171146; doi:10.1038/s41467-020-15606-0)
Supplement: Supplementary file 1 — Supplementary Information [file 41467_2020_15606_MOESM1_ESM.pdf]

Supplementary Information

Molecular profiling of driver events in metastatic uveal melanoma

Karlsson, et al.

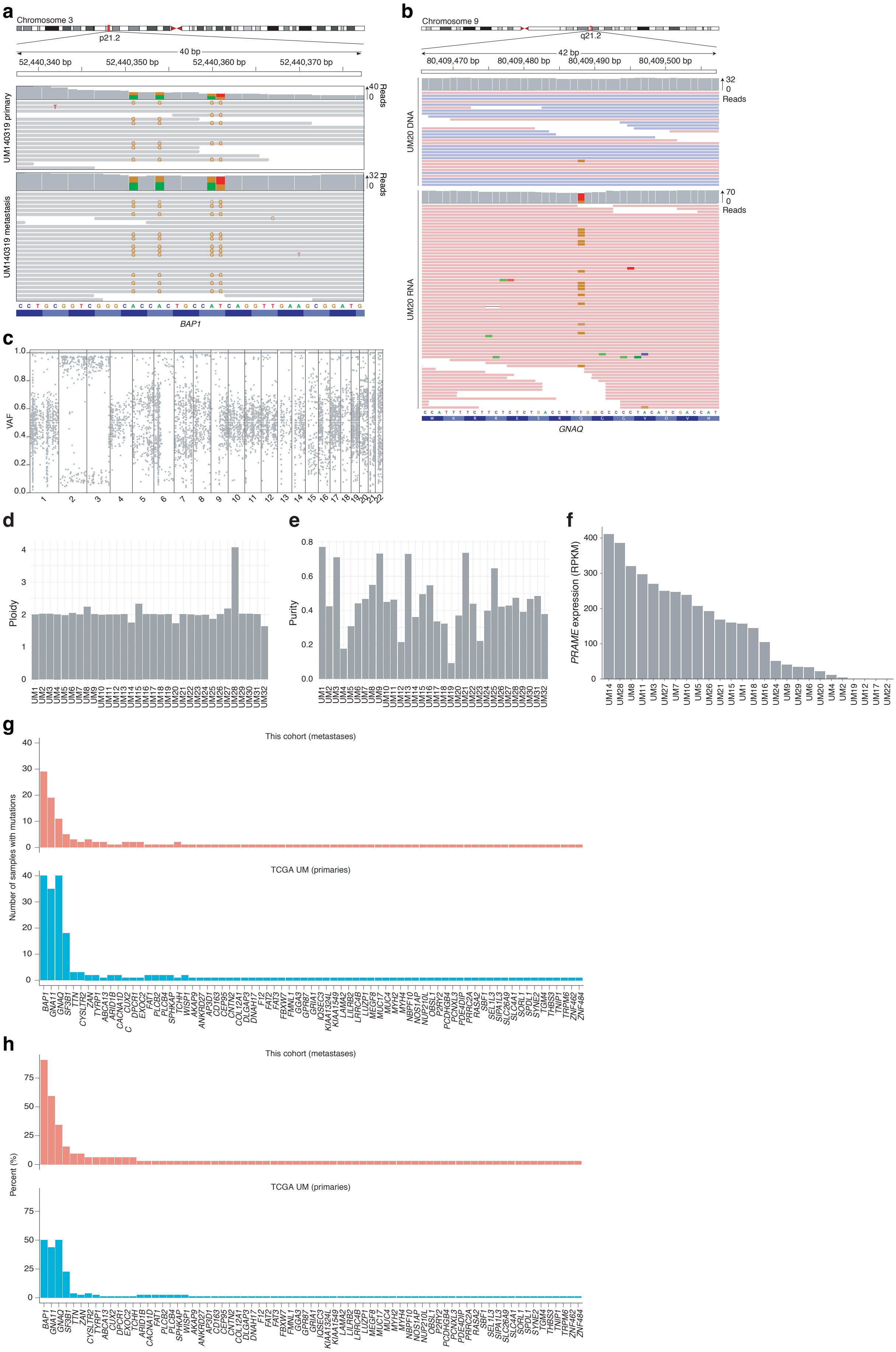

a
Chromosome 3
p21.2
40 bp
52,440,340 bp
52,440,350 bp
52,440,360 bp
52,440,370 bp
UM140319 primary
UM140319 metastasis
Reads
BAP1
b
Chromosome 9
q21.2
42 bp
80,409,470 bp
80,409,480 bp
80,409,490 bp
80,409,500 bp
UM20 DNA
UM20 RNA
Reads
GNAQ
c
VAF
d
Ploidy
e
Purity
f
PRAME expression (RPKM)
g
This cohort (metastases)
TCGA UM (primaries)
Number of samples with mutations
h
This cohort (metastases)
TCGA UM (primaries)
Percent (%)

Supplementary Figure 1: Additional data on genomic alterations. **a)** Multiple *BAP1* mutations in UM16 present in both metastasis and primary tumor. **b)** RNA-seq alignments confirming a *GNAQ* Q209P mutation in UM20 which is only supported by one read on DNA-seq. **c)** Copy number neutral LOH of chromosome 3 in UM22, as shown by shifts in variant allele frequencies of heterozygous exonic single nucleotide variants. **d)** Tumor ploidy and **e)** purity per sample, with values estimated using ichorCNA. **f)** RPKM-normalized expression of *PRAME* in each tumor. **g)** Number of samples with mutations in all genes in our samples (metastases) relative to TCGA UMs (primary tumors). Only those with at least one mutation in TCGA samples are shown. **h)** Frequencies of these mutations relative to cohort size.

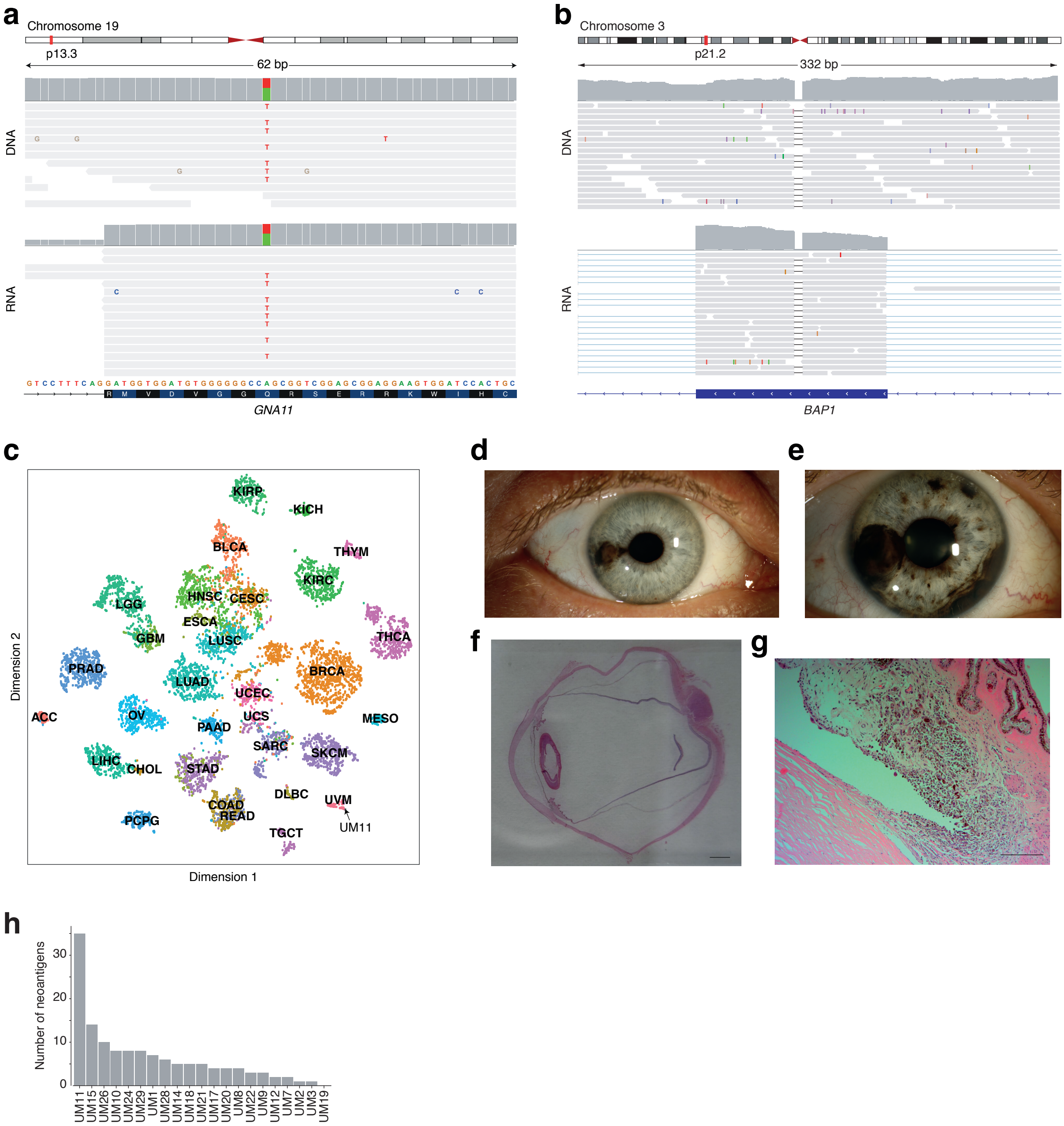

Supplementary Figure 2: Molecular classification of UM11. **a)** *GNA11* Q209L mutation and **b)** *BAP1* deletion in UM11 DNA and RNA. **c)** Transcriptomic classification of UM11 using t-SNE against TCGA tumors (n = 9,583) from 32 cancer types. 6-nearest neighbor classification based on Spearman correlation coefficients gave that 6/6 of the top ranked samples in TCGA were UMs (average correlation coefficient 0.93). **d-g)** Clinical manifestation of an iris melanoma. At diagnosis an iris nevus was seen (d) which progressed to an iris melanoma (e) two years later. **f-g)** Histological sections of different magnifications showing the locally invasive iris melanoma. The H&E staining was done once during clinical routine. In panel (f), the scale bar represents 2 mm. In (g), the scale bar represents 100 μm. **h)** Number of predicted neoantigens per sample, estimated with netMHCpan with HLA genotypes inferred using polysolver.

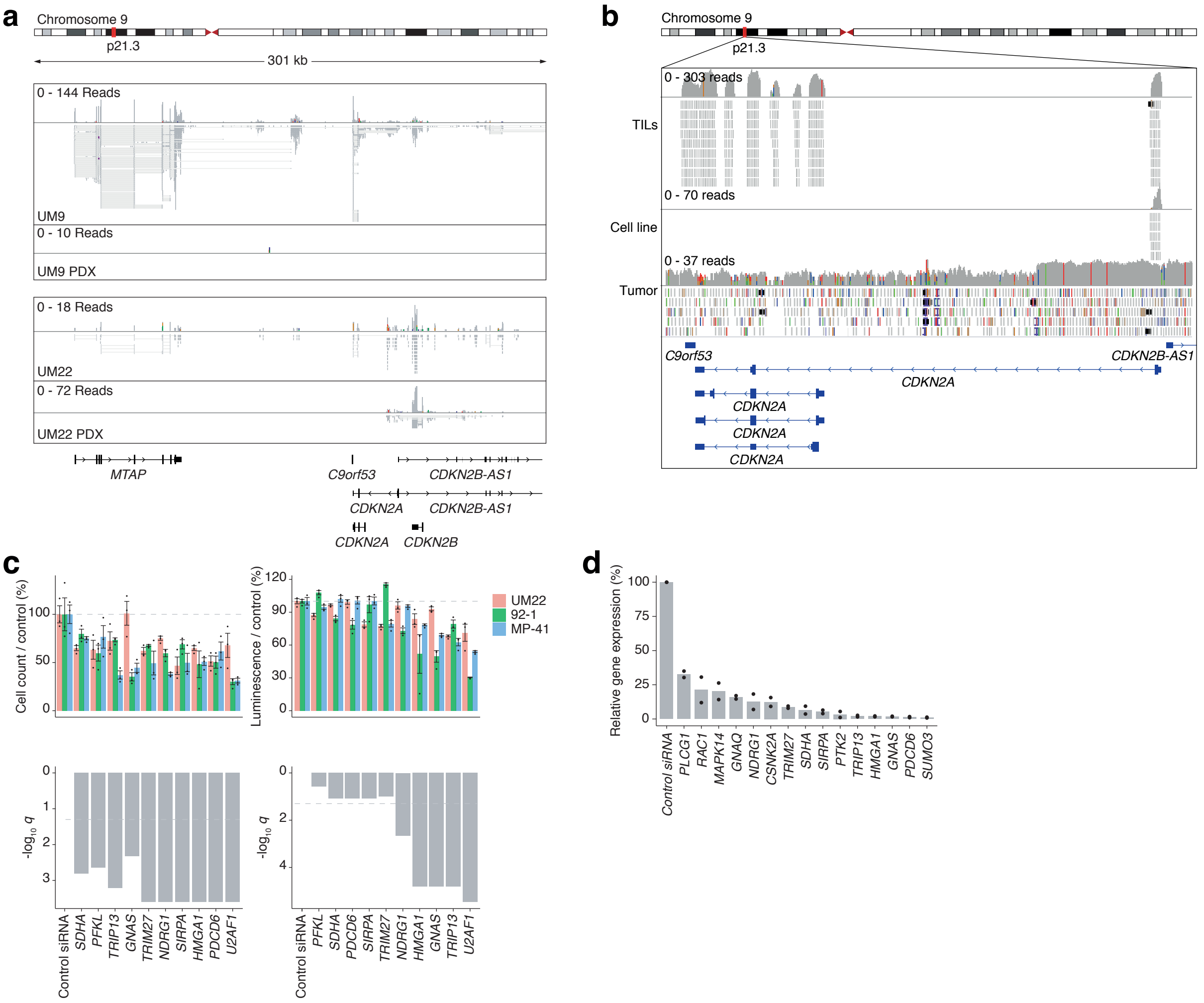

Supplementary Figure 3: Focal deletions of *CDKN2A* and siRNA knockdown experiments**. a)** RNA-seq reads of the *CDKN2A* locus in UM9 and UM22 metastases and PDX models. **b)** Exome sequencing of tumor-infiltrating lymphocytes confirming the somatic identity of *CDKN2A* deletion in UM22. **c)** Functional interrogation by siRNA of a selected set of genes whose expression is elevated due to copy number alterations, similar to **Fig. 2h**, but with secondary candidates. Cells were counted or viability was measured at 72 h, 96h and 96h for the cell lines UM22, MP-41 and 92-1, respectively, after transfection of the siRNA pools. $n = 3$ samples were transfected independently, for each cell line. Data are presented as mean values +/- SEM. Permutation-based two-way ANOVA was used to estimate differences, taking into account both cell line and target gene as variables. q-values were calculates from p-values obtained with this analysis using Benjamini-Hochberg correction, taking into account all genes in **Fig. 2h** as well as those in this panel. This assay was performed in the same experiment as in **Fig. 2h** and the same control is shown here, for comparison. Dotted lines indicate $q < 0.05$. **d)** Estimated siRNA knockdown efficiencies for each gene, with $n = 2$ technical replicates shown.

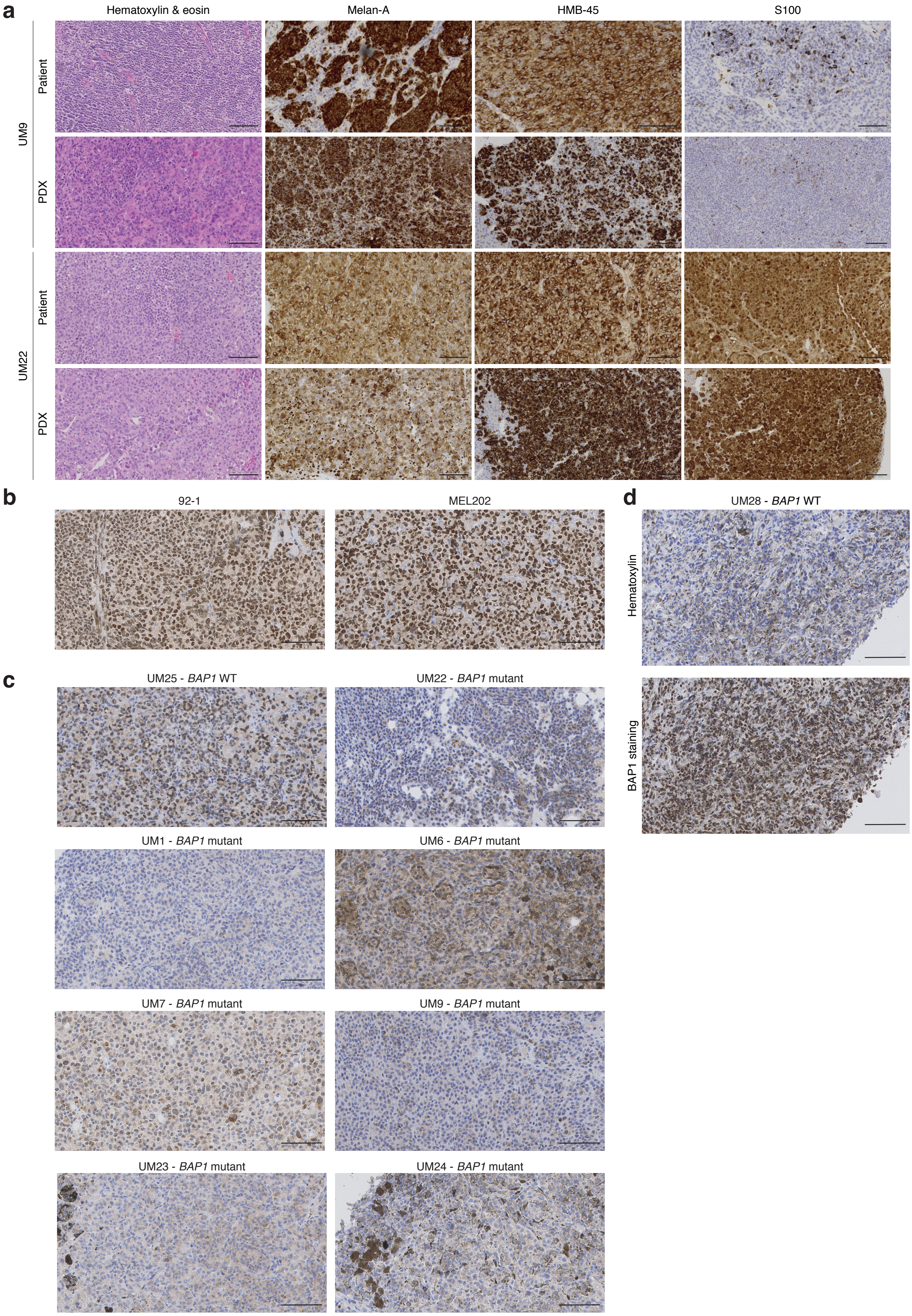

Supplementary Figure 4: Immunohistochemical stainings. **a)** IHC of patient tumors and PDX tumors with respect to hematoxylin and eosin, Melan-A, HMB-45 and S100. **b)** Staining for BAP1 expression

in 92-1 and MEL202, which are *BAP1* wild-type, as reference to (c). **c)** BAP1 staining in PDX models established from eight of the tumors. **d)** BAP1 staining in UM28. Brown deposits in the hematoxylin stained control sample are melanin. In all panels, the stainings were performed once, and for patient samples, in clinical routine. The scale bar represents 100 μm.

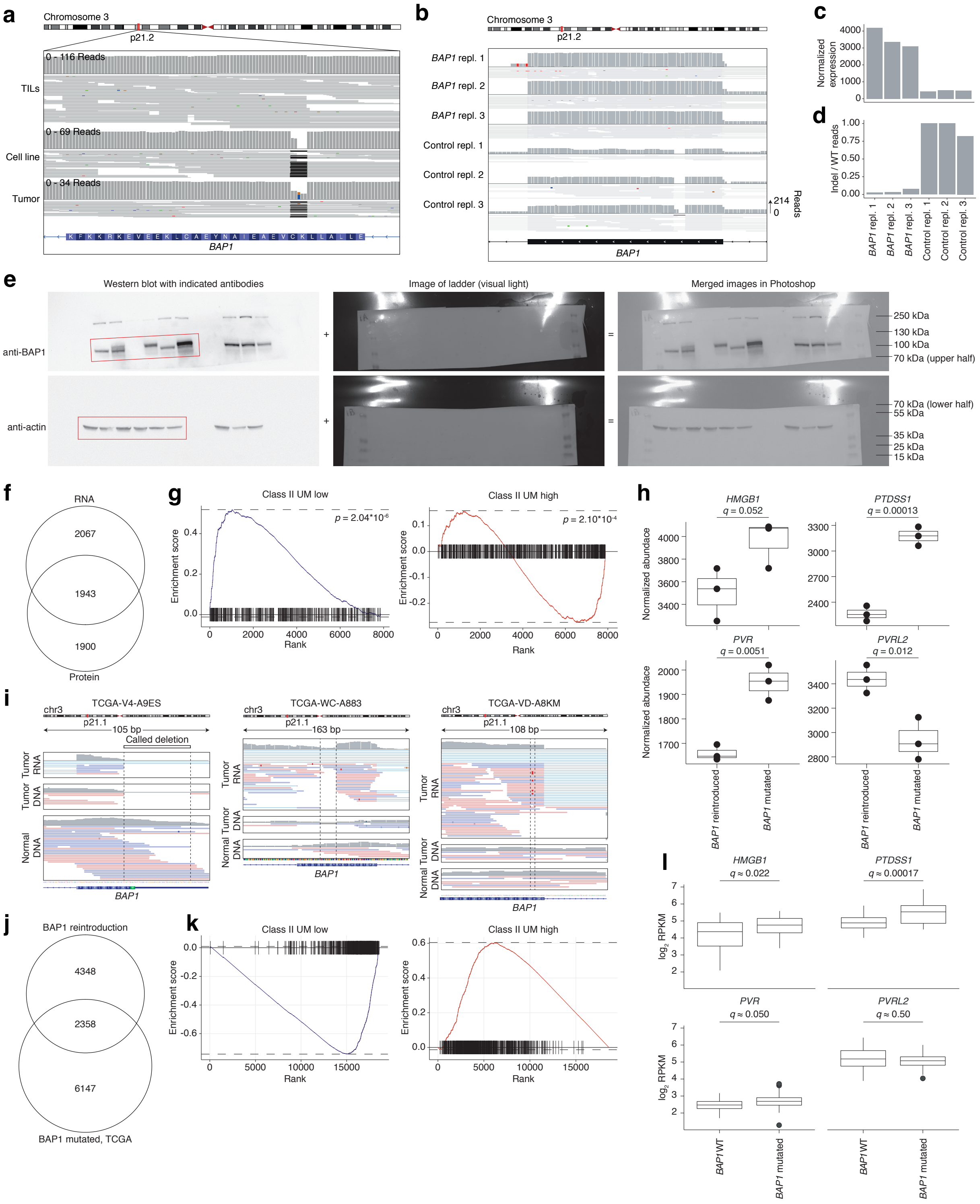

Supplementary Figure 5: *BAP1*-reintroduction. **a)** Reads spanning deletion in the UM22 tumor, normal DNA and the cell line derived from the tumor. **b)** Expression of *BAP1* in control and cell line with reintroduced wild-type *BAP1*. **c-d)** Reads covering the indel region in control and treated condition. **e)** Full western blot images of BAP1 protein expression corresponding to **Fig. 3b** (the red rectangles). Two halves of the same gel are shown and represent one experiment. **f)** Overlap between differentially expressed genes from RNA-seq and differentially expressed proteins from mass spectrometry between *BAP1* mutant UM22 and *BAP1* reintroduced cells, taking into account genes/proteins detected in both experiments. **g)** Gene set enrichment analysis of the categories “Class II UM high” (MSigDB ID: M12490) and “Class II UM low” (M2605). **h)** Differences in protein expression of HMGB1, PTDSS1, PVR and PVRL2 in *BAP1* reintroduced and control cells. *n* = 3 independently

grown samples of cells derived from either the case or control cell lines were used, respectively. **i)** Three likely *BAP1* mutations found by visual inspection in TCGA UM tumors with monosomy 3, which were not previously reported (the deletion in TCGA-V4-A9ES is also present in updated variant calling results from TCGA). **j)** Overlap among genes differentially expressed between *BAP1* mutated and wild-type TCGA tumors and those identified with $q < 0.05$ in the *BAP1*-reintroduction experiment. The intersect represents genes significant in both and with compatible fold changes (high in control cells and high in TCGA *BAP1* mutated samples, and vice versa). **k)** Gene set enrichment analysis with respect to the Class II high/low categories from MSigDB for genes that differed between TCGA samples. **l)** Differences in the expression of *HMGB1*, *PTDSS1*, *PVR* and *PVRL2* in *BAP1* mutated and wild-type TCGA UM samples, representing $n = 40$ biologically independent samples, respectively. In box plots shown, horizontal lines indicate median, lower and upper bounds of boxes represent the first and third quartiles, whiskers represent the smallest/largest data point at most 1.5 times inter-quartile range from the lower and upper bound, respectively, and any points outside of whiskers are individually indicated as outliers. WT: wildtype.

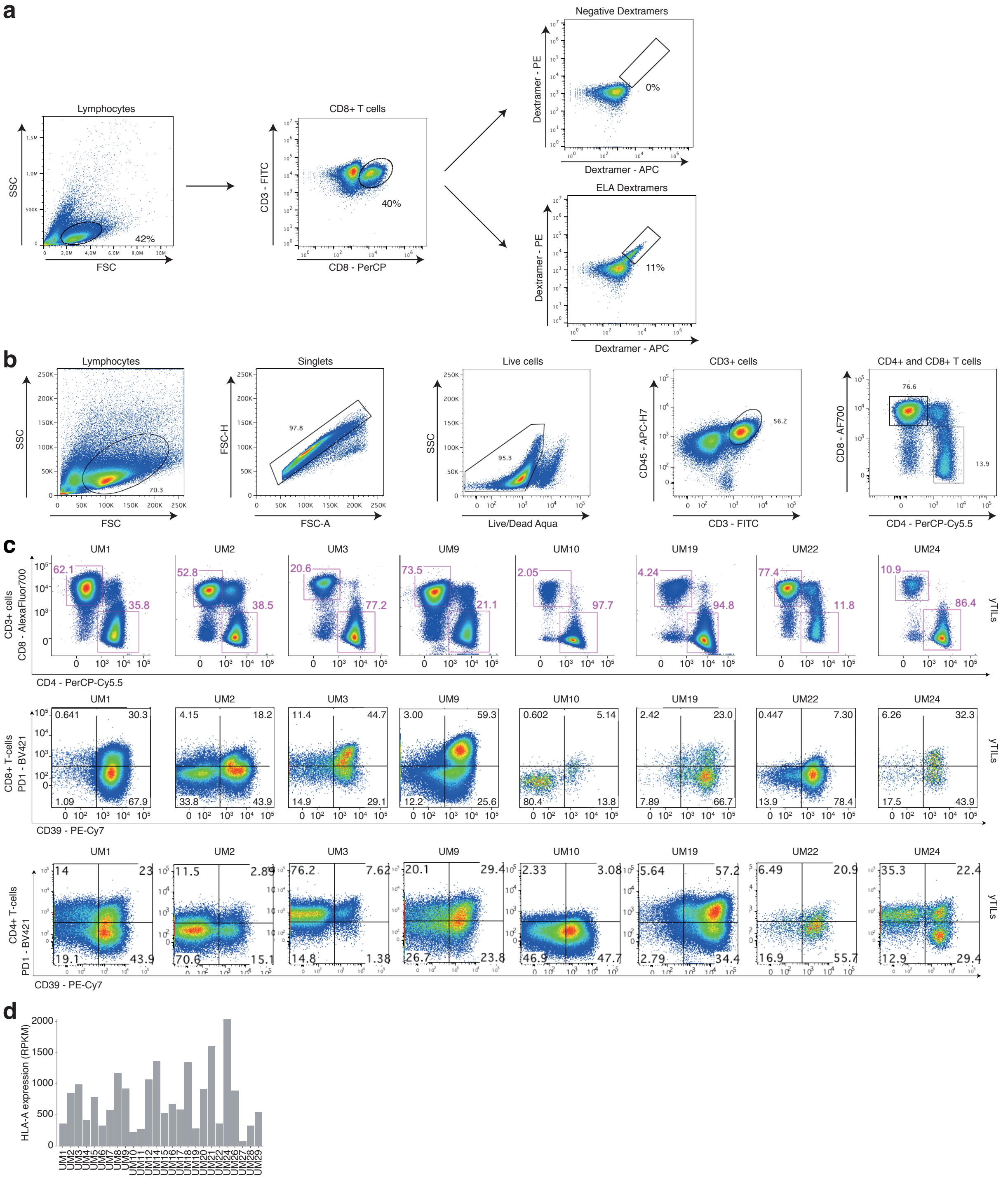

Supplementary Figure 6: Analysis of tumor-infiltrating lymphocytes. **a-b)** Gating strategy for identification of CD4+ and CD8+ T cells among REP-TILs, yTILs and original material from the metastasis. In (a)-(c), the color scale represents relative density, where blue indicates low and red high density. **a)** Representative plots from UM13 showing the strategy used to identify MART-1 specific CD8+ T cells among REP-TILs. **b)** Gating strategy from yTIL material of UM22 illustrating the strategy for analysis of CD4+ and CD8+ T cells in biopsy and yTIL samples. **c)** Flow cytometry analysis of T-cells, with respect to CD3+CD4+ and CD3+CD8+ cells, and proportions of these positive for PD-1 and CD39. **d)** Levels of HLA-A expression in all tumors. REP-TILs: tumor-infiltrating lymphocytes (TILs) expanded with a rapid expansion protocol (high dose of IL2).

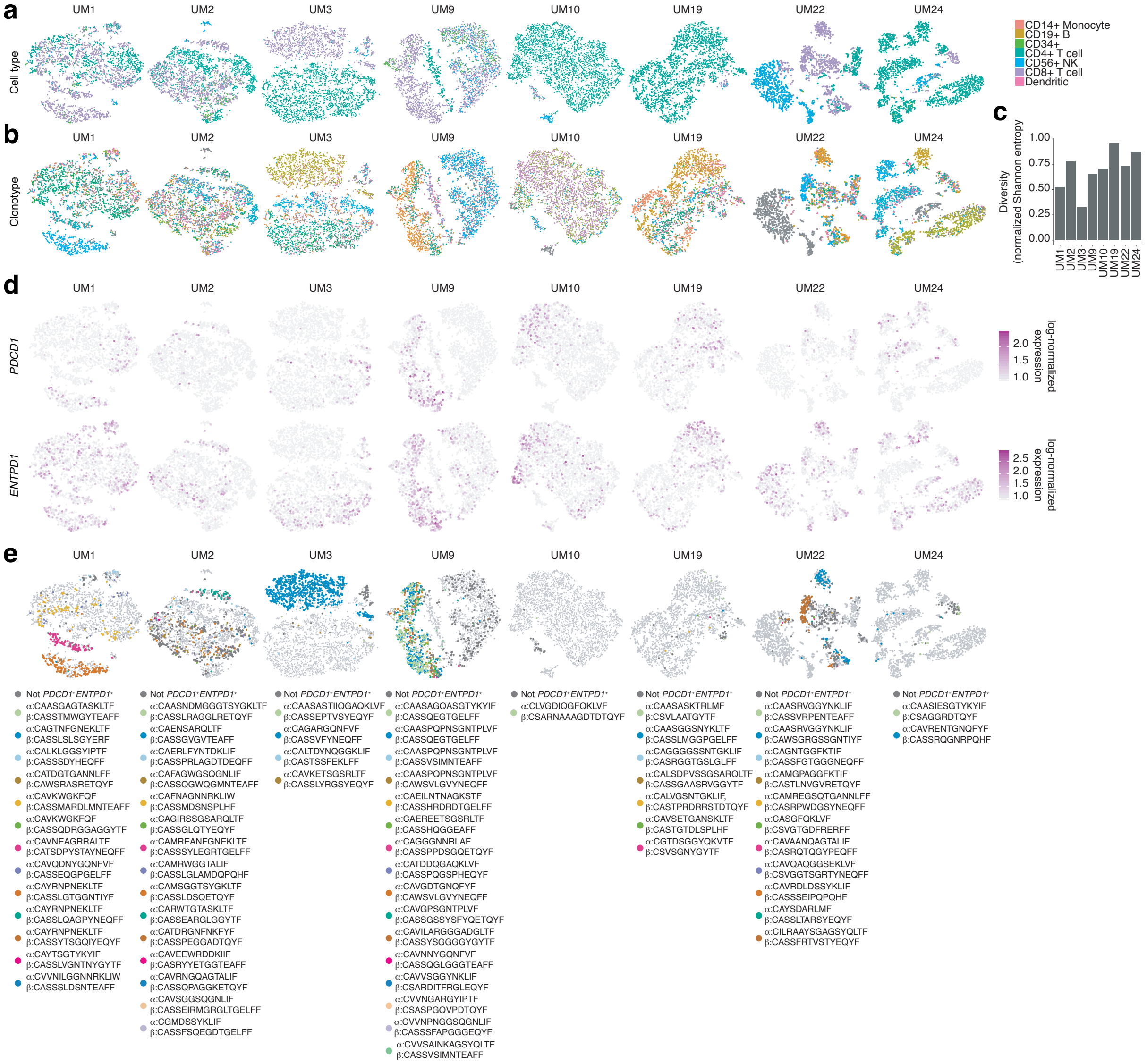

Supplementary Figure 7: Analysis of T cell receptor clonotypes. **a)** t-SNE analysis of single-cell RNA-seq data from yTIL cultures. Cells have been classified according to cell types. **b)** As in a), but colored according to unique TCR clonotypes (separate color schemes for each sample). **c)** Clonotype diversity in each sample, estimated by normalized Shannon entropy calculated on clonotype frequencies. **d)** Expression of *PDCD1* (PD-1) and *ENTPD1* (CD39) per cell, normalized by total molecular counts per sample and then natural log-transformed. **e)** Location of CD8^{+} clonotypes shown in **Fig. 4c** in t-SNE space. Gray indicates clones that were not double positive for *PDCD1* and *ENTPD1* and colors correspond to those in **Fig. 4c**.

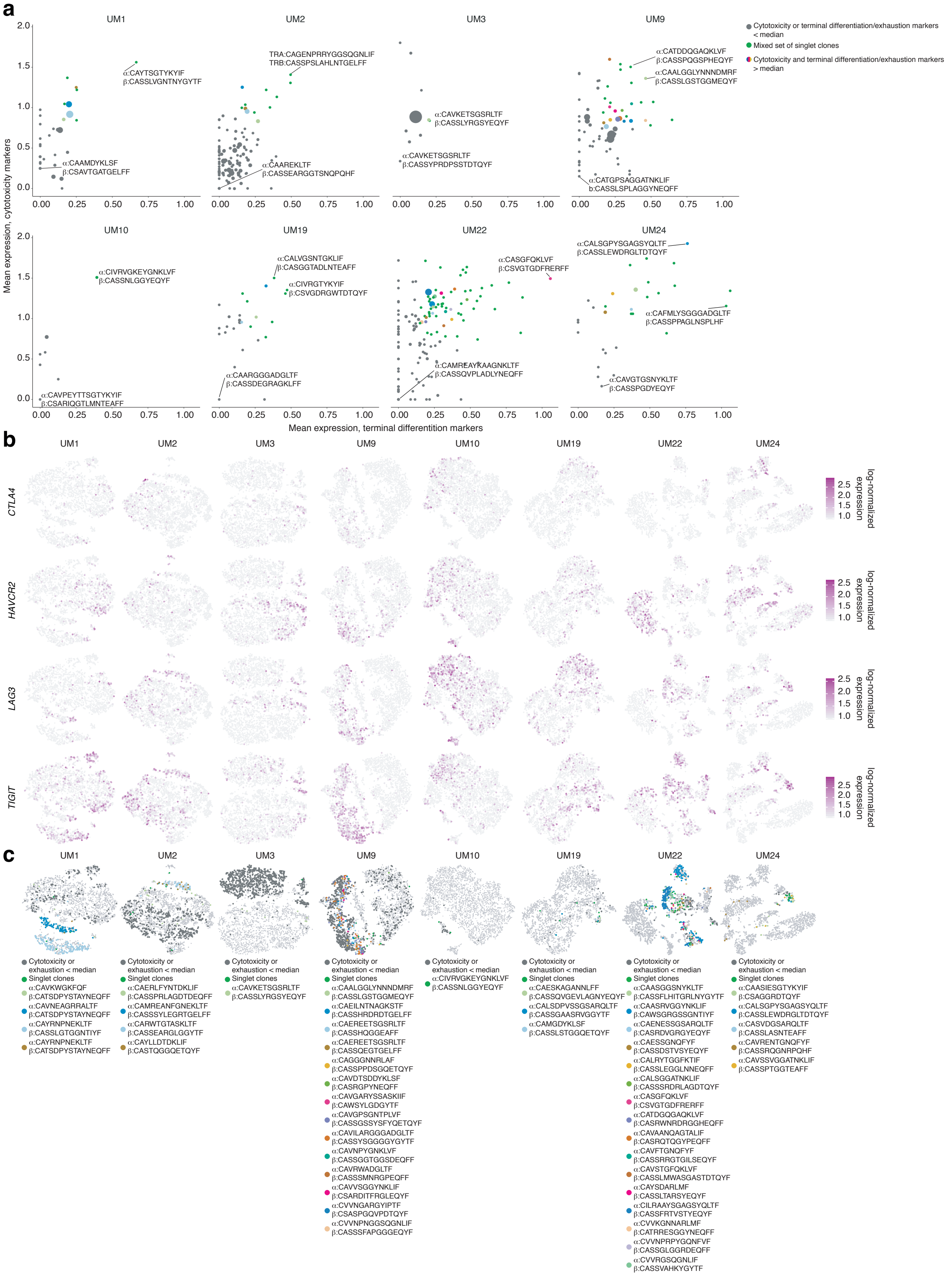

Supplementary Figure 8: Analysis of T cell receptor clonotypes. **a)** Average expression levels of genes in the cytolytics effector pathway (*EOMES*, *TBX21*, *GZMB*, *PRF1*, *FASLG*, *GZMH*, *GZMA*) and terminal differentiation markers (*TIGIT*, *PDCD1*, *CD274*, *CTLA4*, *LAG3*, *HAVCR2*, *CD244*)[1] in clonotypes with one paired alpha and beta chain. Highlighted are clonotypes with the highest and lowest expression in these pathways, respectively. Point sizes are proportional to clonotype frequency.

Gray color indicates clones with less expression than the global average across all samples in either category of genes. Colors correspond to those in (c). **b)** Expression of *CTLA4*, *HAVCR2* (TIM-3), *LAG3* and *TIGIT* per cell, normalized by total molecular counts per sample and then natural log-transformed. **c)** Location of clonotypes shown in (a) in t-SNE space, with corresponding colors. Any color other than gray indicates greater than average expression of markers in both categories.

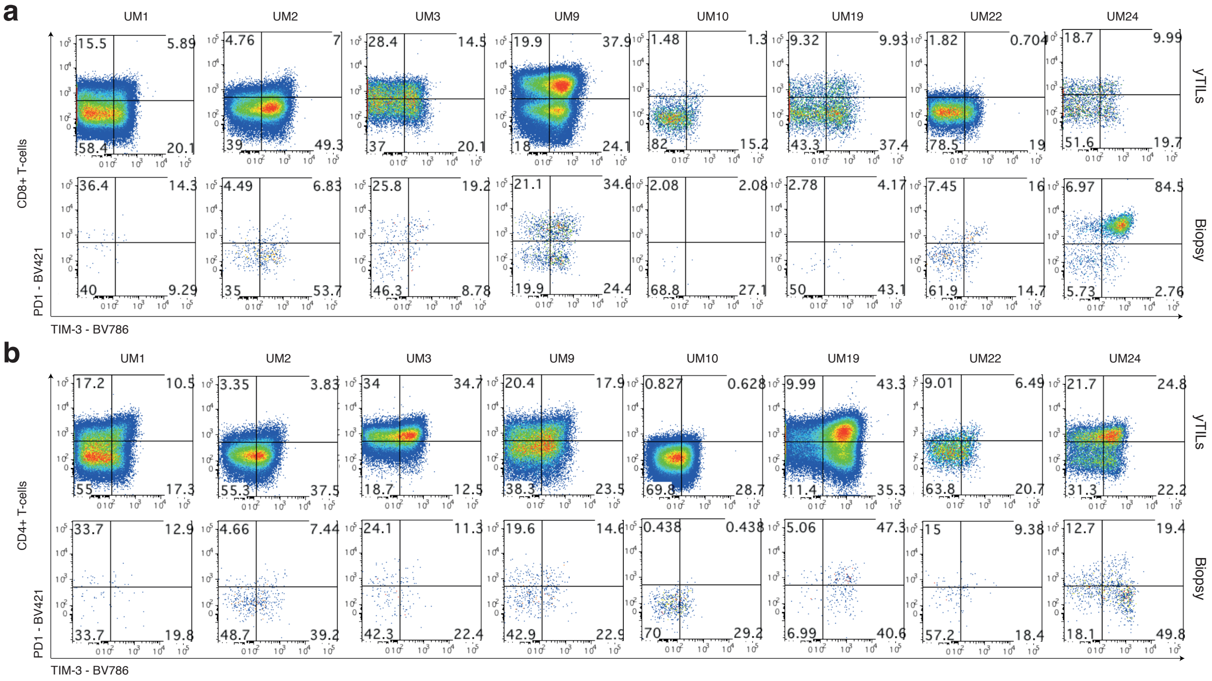

Supplementary Figure 9: Flow cytometry analysis of T-cells. **a)** Proportions of $CD8^+$ T cells positive for PD-1 and TIM-3, as determined by flow cytometry. **b)** As in a), for $CD4^+$ T cells. The color scale represents relative density, where blue indicates low and red high density.

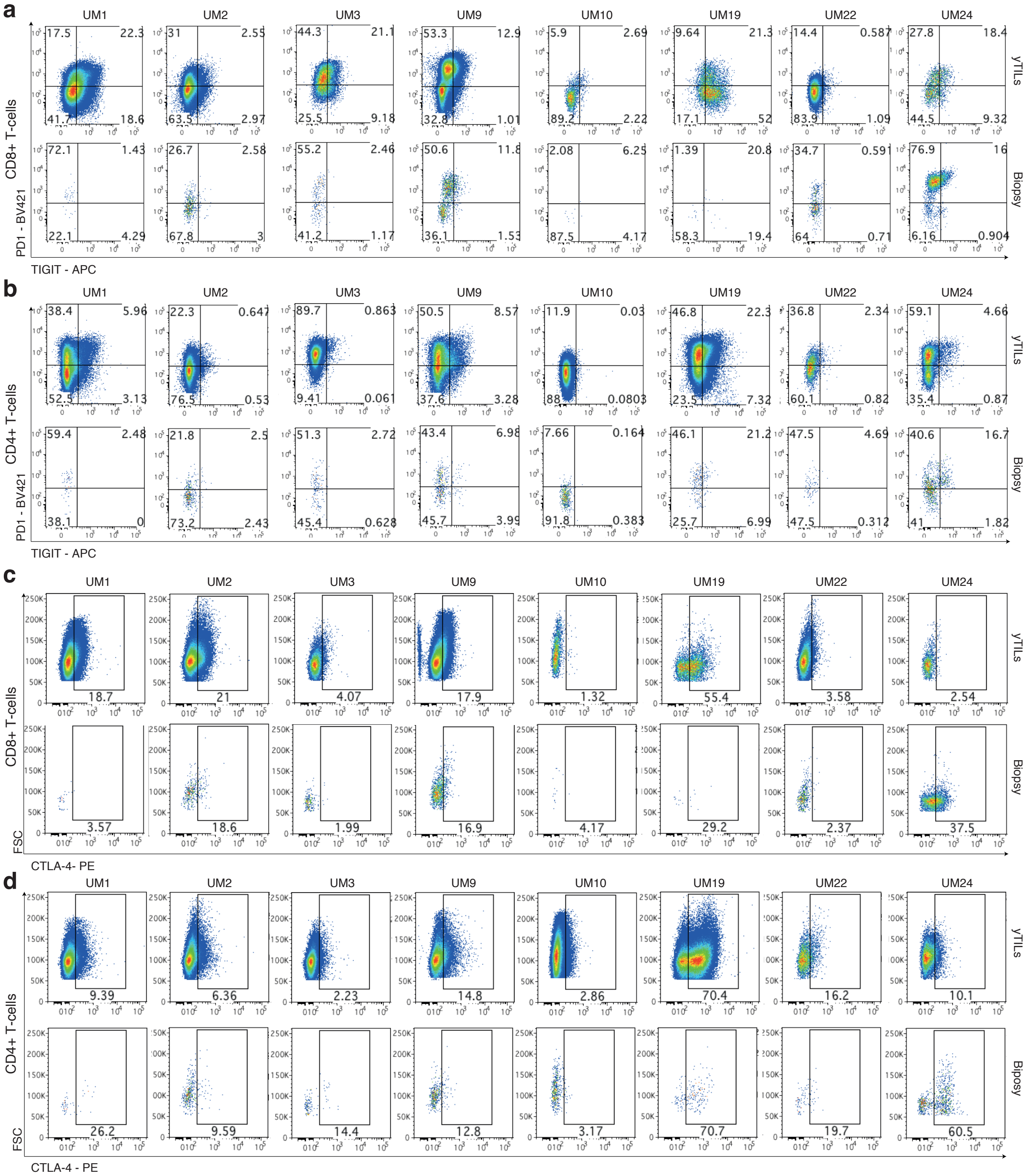

Supplementary Figure 10: Flow cytometry analysis of T-cells. Proportions of **a)** CD8^{+} and **b)** CD4^{+} cells positive for PD-1 and TIGIT. Proportions **c)** CD8^{+} and **d)** CD4^{+} cells positive for CTLA-4. The color scale represents relative density, where blue indicates low and red high density.

Supplementary Table 1: Clinical details of samples.

Sample ID	Metastatic site	Primary site	Sex	Age
UM1	Liver	Choroid	M	73
UM2	Liver	Choroid	M	72
UM3	Liver	Ciliary	F	34
UM4	Liver	Choroid	M	60
UM5	Liver	Choroid	F	71
UM6	Subcutaneous	Choroid	F	77
UM7	Subcutaneous	Choroid	F	64
UM8	Liver	Unknown	F	58
UM9	Liver	Choroid	M	60
UM10	Liver	Choroid	M	64
UM11	Liver	Iris	M	58
UM12	Liver	Choroid	F	61
UM13	Liver	Choroid	M	64
UM14	Liver	Unknown	F	43
UM15	Subcutaneous	Choroid	F	71
UM16	Liver	Choroid	F	72
UM17	Liver	Choroid	F	65
UM18	Liver	Choroid	M	68
UM19	Liver	Choroid	F	64
UM20	Liver	Choroid	M	76
UM21	Liver	Choroid	F	63
UM22	Subcutaneous	Choroid	F	54
UM23	Liver	Choroid	M	58
UM24	Liver	Choroid	M	71
UM25	Liver	Choroid	F	74
UM26	Subcutaneous	Choroid	F	76
UM27	Subcutaneous	Choroid	M	66
UM28	Liver	Choroid	F	71
UM29	Liver	Choroid	M	46
UM30	Liver	Choroid	F	68
UM31	Liver	Choroid	M	80
UM32	Liver	Choroid	F	66

Supplementary References

1. Azizi, E. *et al.* Single-Cell Map of Diverse Immune Phenotypes in the Breast Tumor Microenvironment. *Cell* **174**, 1293-1308.e36 (2018).
