# Supplementary material for: Molecular profiling of driver events in metastatic uveal melanoma
Source: Nat Commun. 2020 Apr 20;11:1894. doi: 10.1038/s41467-020-15606-0 (PMC7171146; doi:10.1038/s41467-020-15606-0)
Supplement: Supplementary file 3 — Description of Additional Supplementary Files [file 41467_2020_15606_MOESM3_ESM.docx]

Description of Additional Supplementary Files

**Supplementary Data 1:** List of mutations. Annotated somatic mutations detected in each tumor.

**Supplementary Data 2:** Associations between copy number alterations and metastasis. Statistics for tests of association between broad copy number changes in our samples versus TCGA tumors.

**Supplementary Data 3:** Ranked genes in copy number alterations. Ranking of genes relevant in broad copy number changes, per affected chromosome arm. The data sheets include genes ordered by presence of correlation between copy number and expression in both datasets, known protein-protein interactions and univariate survival statistics; results of enrichment analysis for MSigDB “canonical pathway” gene sets among top ten ranked genes in the combined ranked lists in either regions of gain or loss; additional statistics for siRNA knockdown experiments of candidate genes. All tests were two-sided, except for enrichment tests, which were one-sided (the MSigDB algorithm does not assess depletion).

**Supplementary Data 4:** Differentially expressed genes after *BAP1* reintroduction. Differentially expressed genes between cells transduced with functional *BAP1* vectors or empty vectors. *n* = 3 independently grown samples of cells derived from either the case or control cell lines were used, respectively. All tests were two-sided.

**Supplementary Data 5:** Gene set enrichment analysis. Gene set enrichment analysis for the chemical and genetic perturbations category from MSigDB of genes assessed in the comparison of replicates transduced with functional *BAP1* vectors or empty vectors.

**Supplementary Data 6:** Differentially expressed proteins after *BAP1* reintroduction. Normalized abundances and fold-changes of proteins detected by mass spectrometry in *BAP1*-reintroduced UM22 cells and empty vector-treated control cells. All tests were two-sided.

**Supplementary Data 7:** HLA genotypes. HLA genotypes inferred by polysolver based on whole-genome sequencing data, and OptiType, from RNA-seq data, of all tumors.
