# Supplementary material for: Molecular profiling of driver events in metastatic uveal melanoma
Source: Nat Commun. 2020 Apr 20;11:1894. doi: 10.1038/s41467-020-15606-0 (PMC7171146; doi:10.1038/s41467-020-15606-0)
Supplement: Supplementary file 11 — Reporting Summary [file 41467_2020_15606_MOESM11_ESM.pdf]

nature research

Corresponding author(s): Jonas A. Nilsson

Last updated by author(s): 14/3/20

Reporting Summary

Nature Research wishes to improve the reproducibility of the work that we publish. This form provides structure for consistency and transparency in reporting. For further information on Nature Research policies, see Authors & Referees and the Editorial Policy Checklist .

Statistics

For all statistical analyses, confirm that the following items are present in the figure legend, table legend, main text, or Methods section.

n/a	Confirmed	
☐	☒	The exact sample size (*n*) for each experimental group/condition, given as a discrete number and unit of measurement
☐	☒	A statement on whether measurements were taken from distinct samples or whether the same sample was measured repeatedly
☐	☒	The statistical test(s) used AND whether they are one- or two-sided<br>*Only common tests should be described solely by name; describe more complex techniques in the Methods section.*
☐	☒	A description of all covariates tested
☐	☒	A description of any assumptions or corrections, such as tests of normality and adjustment for multiple comparisons
☐	☒	A full description of the statistical parameters including central tendency (e.g. means) or other basic estimates (e.g. regression coefficient) AND variation (e.g. standard deviation) or associated estimates of uncertainty (e.g. confidence intervals)
☐	☒	For null hypothesis testing, the test statistic (e.g. *F*, *t*, *r*) with confidence intervals, effect sizes, degrees of freedom and *P* value noted<br>*Give P values as exact values whenever suitable.*
☒	☐	For Bayesian analysis, information on the choice of priors and Markov chain Monte Carlo settings
☐	☒	For hierarchical and complex designs, identification of the appropriate level for tests and full reporting of outcomes
☐	☒	Estimates of effect sizes (e.g. Cohen's *d*, Pearson's *r*), indicating how they were calculated

Our web collection on statistics for biologists contains articles on many of the points above.

Software and code

Policy information about availability of computer code

Data collection	TCGAbiolinks (v. 2.15.2), Proteome Discoverer (v. 2.2), FlowJo (v. 10.6.1).
Data analysis	bwa (v. 0.7.12), GATK (v. 3.3.0, v. 4.0.11.0), Picard (v. 1.109), Disambiguate (v. 2018.05.03), STAR (v. 2.7.1a), htseq-count (HTSeq v. 0.6.0), MuTect 2 (GATK v. 4.0.11.0), VEP (v. 91.3), ANNOVAR (v. 2016-05-11), R (v. 3.6.0), MutationalPatterns (v. 1.10.0), hisat (v. 0.1.6-beta), polysolver (v. 1.0), OptiType (v. 1.3.2), netMHCpan (v. 4.0), kallisto (v. 0.44.0), binocular (v. 0.2), ichorCNA (v. 0.1.0), CNVkit (v. 0.9.6a0), edgeR (v. 3.28.0), biomaRt (v. 2.42.0), survival (v. 3.1-8), limma (v. 3.40.6), iRefR (v. 1.13), DESeq2 (v. 1.22.1), fgsea (v. 1.10.1), MATLAB (v. R2018a), Rtsne (v. 0.15), Mascot (v. 2.5.1), Cellranger (v. 2.1.1), Seurat (v. 3.1.3), DoubletFinder (v. 2.0.2), diverse (v. 0.1.5). Statistical analyses were performed in R. Code is available at https://bitbucket.org/jowkar/um.

For manuscripts utilizing custom algorithms or software that are central to the research but not yet described in published literature, software must be made available to editors/reviewers. We strongly encourage code deposition in a community repository (e.g. GitHub). See the Nature Research guidelines for submitting code & software for further information.

Data

Policy information about availability of data

All manuscripts must include a data availability statement. This statement should provide the following information, where applicable:
- Accession codes, unique identifiers, or web links for publicly available datasets
- A list of figures that have associated raw data
- A description of any restrictions on data availability

Whole-genome, exome and RNA sequence data generated for this study has been deposited at the European Genome-Phenome Archive, which is hosted by the EBI and the CRG under accession numbers EGAS00001004296 [https://ega-archive.org/studies/EGAS00001004296] and EGAS00001003026 [https://ega-archive.org/studies/EGAS00001003026]. Single-cell transcriptomics and TCR data is available at ArrayExpress with the identifier E-MTAB-8846 [https://www.ebi.ac.uk/arrayexpress/experiments/E-MTAB-8846]. TCGA data used in this study is available from the GDC Data Portal (https://portal.gdc.cancer.gov/) under restrictions of controlled access for raw data. Mass spectrometry proteomics data generated for this study have been deposited to the ProteomeXchange Consortium via the

PRIDE partner repository with the dataset identifier PXD017743 [https://www.ebi.ac.uk/pride/archive/projects/PXD017743]. Figures with associated raw data are Fig. 1-4, Supplementary Fig. 1-3 and Supplementary Fig. 5-8. Mutation data from the COSMIC database can accessed at https://cancer.sanger.ac.uk/cosmic/download and mutational signature data at http://cancer.sanger.ac.uk/cancergenome/assets/signatures_probabilities.txt. Data from the Human protein reference database (HPRD) was accessed through the iRefR R package and the database can be downloaded at http://hprd.org/download. Reference protein data from SwissProt was accessed through the Mascot software and the database can be downloaded from https://www.uniprot.org/downloads. Databases formatted for use with ANNVOAR, including ESP6500 and 1000 Genomes, can be downloaded by following the instructions at http://annovar.openbioinformatics.org/en/latest/user-guide/download/. Data from the Genome Aggregation database (gnomAD) can be accessed at ftp://ftp.broadinstitute.org/bundle/Mutect2/af-only-gnomad.raw.sites.b37.vcf.gz.

Field-specific reporting

Please select the one below that is the best fit for your research. If you are not sure, read the appropriate sections before making your selection.

- [x] Life sciences
- [ ] Behavioural & social sciences
- [ ] Ecological, evolutionary & environmental sciences

For a reference copy of the document with all sections, see nature.com/documents/nr-reporting-summary-flat.pdf

Life sciences study design

All studies must disclose on these points even when the disclosure is negative.

Sample size	Tumors from 32 patients were studied. The majority of the patients were previously enrolled in a clinical trial, although the present study does not intend to describe the outcome of that trial, but rather to genomically profile the tumors. The tumors were included based on availability of material. Although a greater number of tumors would provide added power for new discoveries, interesting findings could potentially also arise from a single individual. Therefore, prior sample size calculations were not applicable in this case.
Data exclusions	No patients were excluded from the study. Some analyses required paired genomic and transcriptomic data, the latter of which was not available for two samples, leading to their exlusion from those analyses. In a single-cell analysis comprising a large number of cells, some were excluded due to quality issues, as defined by default settings in the tool (CellRanger) used for pre-processing.
Replication	Three replicates were used to test for differences in cells with reinserted functional BAP1. RNA-seq alignments, Western blot, immunohistochemsitry and proteomics confirmed sucessful viral reintroduction in all three. Genes of interest differing between the conditions with and without this gene reintroduced were successfully validated on subsequent RT-qPCR-experiments, also using three replicates. 6-nearest neighbor transcriptomic classification of a specific tumor was carried out against a 9583 tumors from TCGA, and all six top ranking correlating tumors agreed on a single classification. The method was complemented by mutation analysis of DNA and RNA from the same sample, together sucessfully ruling out a sample mixup. In a study of genes of relevance in recurrent broad copy number changes, genes of interest were compared between datasets from ours and two other studies, validating many of the ranking as top candidates. In assays to test cell proliferation and viability effects upon siRNA knockdown of candidate genes, three biological replicates were used.
Randomization	No predefined experimental groups were used in this study.
Blinding	Animal experiments were performed by a technician that was blinded to the experimental design. qRT-PCR analyses were not blinded since they were screens to identify potentially important genes. The genes identified did not have any bearing on the overall interpretation of the data so blinding was not needed. The BAP1 qRT-PCR analysis was performed by two different operators yielding the same results, one of which was blinded to the results from the RNA-seq data.

Reporting for specific materials, systems and methods

We require information from authors about some types of materials, experimental systems and methods used in many studies. Here, indicate whether each material, system or method listed is relevant to your study. If you are not sure if a list item applies to your research, read the appropriate section before selecting a response.

Materials & experimental systems

n/a	Involved in the study	
[ ]	[x]	Antibodies
[ ]	[x]	Eukaryotic cell lines
[x]	[ ]	Palaeontology
[ ]	[x]	Animals and other organisms
[ ]	[x]	Human research participants
[x]	[ ]	Clinical data

Methods

n/a	Involved in the study	
[x]	[ ]	ChIP-seq
[ ]	[x]	Flow cytometry
[x]	[ ]	MRI-based neuroimaging

Antibodies

Antibodies used	The following antibodies were used for surface staining: CD3 (clone: HIT3a, catalog: 300306, lot: B274310, dilution: 1:200), CD4 (clone: A161A1, catalog: 357414, lot: B238830, dilution: 1:200), CD8 (clone: HIT8a, catalog: 300920, lot: B256905, dilution: 1:200), CD45 (clone: 2D1, catalog: 368516, lot: B251494, dilution: 1:200), CD69 (clone: FN50, catalog: 310933, lot: B251799,

dilution: 1:200), CTLA4 (clone: BNI13, catalog: 369603, lot: B242857, dilution: 1:100), PD-1 (clone: EH12.H7, catalog: 329920, lot: B255122, dilution: 1:200), TIGIT (clone: A15153G, catalog: 747839, lot: 8267685, dilution: 1:100) and TIM3 (clone: F38-2E2, catalog: 345031, lot: B241708, dilution: 1:100) from BioLegend and CD39 (clone: eBioA1, catalog: 25-0399-41, lot: 4329374, dilution: 1:200) from eBioscience. Antibodies for IHC were: anti-PMEL (clone: HMB45, catalog: GA05261-2, ready-to-use solution for Autostainers), Melan A (clone A103, catalog: IS63330-2, ready-to-use solution), and anti-S100 (clone: IR504, catalog: IS50430-2, ready-to-use solution), from Agilent/Dako. BAP1 antibody was purchased from Santa Cruz (clone: C-4; catalog: sc-28383), used with dilution 1:1000 for Western blot and 1:100 for IHC. Beta-actin antibody used for Western blot was purchased from Sigma (clone AC-15, catalog: A1978, dilution 1:10000).

Validation

All flow cytometry antibody clones were well-established clones sold by two or more companies. Antibodies were titrated in-house and validated by flow cytometry at the indicated dilution factors. For IHC, antibodies used were validated at the Clinical pathology lab of Sahlgrenska Hospital by staining tissue or tumor slides known to be positive or negative for the proteins. The stainings presented were performed in parallell with clinical routine stainings using an Autostainer. The Western blot antibodies were validated based on known sizes of the BAP1 and beta-actin proteins (Supplemental Figure 5e).

Eukaryotic cell lines

Policy information about cell lines

Cell line source(s)

The cell line was generated from a tumor that grew up in a PDX model (UM22). Its validation by exome sequencing and RNA sequencing is described in the manuscript.

Authentication

As described in the manuscript, the cell line and original tumor were both sequenced.

Mycoplasma contamination

Mycoplasma control was performed regularly using a PCR method. Only mycoplasma negative cell lines were used in the study.

Commonly misidentified lines
(See ICLAC register)

None

Animals and other organisms

Policy information about studies involving animals; ARRIVE guidelines recommended for reporting animal research

Laboratory animals

The mice were housed in the pathogen-free animal facility of University of Gothenburg. Mice were kept in cages with individual ventilation at ambient temperature (21-23 degrees Celsius) and 20-40% humidity. Mice were given free access to food and water. The dark-ligh cycle was 12 hrs dark and light, respectively (dark 7pm to 7am). 6-8 week old female NOD/SCID/Il2R-gamma knockout mice (NOG/NSG) mice were used in the experiments.

Wild animals

No wild animals were used.

Field-collected samples

No field-collected samples were used.

Ethics oversight

Regional animal ethics committee of Gothenburg approval #36-2014.

Note that full information on the approval of the study protocol must also be provided in the manuscript.

Human research participants

Policy information about studies involving human research participants

Population characteristics

32 metastatic tumors from patients diagnosed with uveal melanoma, six subcutaneous and 26 from the liver, were collected from 14 males and 18 females with an average age of 65 years and a range of 34-80. Twenty-eight of the corresponding primary tumors were pathologically designated as originating from the choroid, one from the ciliary body, one from the iris, and two unknown. All liver biopsies came from patients that were untreated at the time of biopsy and eighteen of them had been enrolled in the SCANDIUM phase III clinical trial of IHP. All cutaneous metastases came from patients that had been treated with chemotherapy (IHP, dacarbazine and/or taxanes).

Recruitment	The trial from which most samples were derived is open for patients from all of Scandinavia. Patients need to have a confirmed diagnosis of uveal melanoma with liver metastases only. No extra-hepatic metastases are allowed. Patients are >18 yrs of age and ECOG status 0-1. Only patients fit to handle perfusion (based on liver values) are included. The study is first-line and no other previous treatment is allowed.<br>The samples received were subjected to RNA/DNA preparation and only samples fullfilling the criteria of high integrity RNA/DNA were included. No knowledge about the trial participants were known before sequencing and no samples were excluded on any other grounds than experimental failure (RNA/DNA preparation or post-sequencing QC). Therefore no potential self-selection bias were present in the study. Other biases that could impact the study was the site of metastasis. Only patients with liver metastases or subcutaneous metastases were included because of access to surgically resected material. Hence the study does not address genomic driver events of uveal melanoma metastatic to other sites. However, liver and/or subcutaneous metastases is present in 95% of all patients, suggesting that our sample collection is highly representative of the most common forms of uveal melanoma metastases. For single-cell immune analyses, we could only sequence tumors with high enough immune infiltrates, meaning that potentially interesting cases of immune cell exclusion might be missed. One of the immune-profiled tumors came from a patient with previous immunotherapy, which could potentially influence immune-infiltrates, which is noted in the relevant figure legends.
Ethics oversight	The patients received oral and written information and signed the informed consent agreement according to the ethical approval at the Göteborg Human ethical review board (approval #289-12, #144-13 and #44-18).

Note that full information on the approval of the study protocol must also be provided in the manuscript.

Flow Cytometry

Plots

Confirm that:

- [x] The axis labels state the marker and fluorochrome used (e.g. CD4-FITC).
- [x] The axis scales are clearly visible. Include numbers along axes only for bottom left plot of group (a 'group' is an analysis of identical markers).
- [x] All plots are contour plots with outliers or pseudocolor plots.
- [x] A numerical value for number of cells or percentage (with statistics) is provided.

Methodology

Sample preparation	Pieces of biopsies of metastases were processed by mechanical dissociation with scalpel blades followed by filtration through a cell strainer. Cells were cryopreserved until use. Young TILS (yTILs) were made from other pieces of the biopsies. These pieces were cultured in wells in medium containing human serum and IL-2. After 14-21 days of culture, yTILs had grown out of the pieces and established a yTIL culture which was strained and cryopreserved until use in flow cytometric analyses.
Instrument	BD Accuri C6 (BD Biosciences), BD LSRFortessa X20 (BD Biosciences)
Software	BD Accuri C6 Analysis 2 (BD Biosciences), FACSDiva Software Version 8.0.1 (BD Biosciences), FlowJo Version 9.9.6 (FlowJo, LLC), FlowJo Version 10.5.3 (FlowJo, LLC)
Cell population abundance	No sorted cells were used in the present study.
Gating strategy	Lymphocytes were identified by conventional FSC vs SSC gating followed by FSC-H vs FSC-A to exclude doublets. For identification of dextramer binding cells we used CD3 staining to exclude rare non-T cell events and CD8 staining to separate CD8 T cells from CD4 T cells. Dextramers carrying HLA-A2:01 loaded with an irrelavant peptide were used as negative control and CD8 T cells showing both binding to PE and APC labelled dextramers were considered TCR specific for the loaded peptide noted in the figure.<br>For CD4 and CD8 T cell analysis in biopsy or in yTIL material, dead cells were excluded by Live/Dead Aqua fluorescence and T cells were identified by co-staining of CD3 and CD45 to discriminate non-haematopoietic cells and non-T cells. From the CD3+ CD45+ population CD4 and CD8 staining was used to separate CD4+ and CD8+ T cells, double positive and double negative events were discriminated.<br>For analysis of PD-1, TIGIT, TIM-3, CD39 and CTLA-4 gates determining postive events were set according to population density in samples were positive events could could clearly be distinguished from negative events.

- [x] Tick this box to confirm that a figure exemplifying the gating strategy is provided in the Supplementary Information.
